# Will Women Interact with Technology to Understand Their Cardiovascular Risk and Potentially Increase Activity?

**DOI:** 10.1089/biores.2018.0047

**Published:** 2019-07-03

**Authors:** Kathy Hildebrand, Kathryn King-Shier, Lorraine Venturato, Christy Tompkins-Lane

**Affiliations:** ^1^ Faculty of Nursing, University of Calgary, Calgary, Canada.; ^2^ Mount Royal University, Calgary, Canada.

**Keywords:** activity, cardiovascular risk, wearable technology, women

## Abstract

Cardiovascular disease (CVD) continues to be one of the leading causes of death for women. New approaches need to be identified that will enable women to recognize modifiable risk factors and target their efforts toward prevention. The objectives of this study were to (1) determine if women would access Vivametrica^™^ to assess CVD risk, (2) identify whether women would increase their physical activity as measured by their daily step counts, and (3) elicit women's opinions about using the system, prospective observational study design.

Thirty-six English-speaking women aged 45–64 years of age, without physical disability, were recruited. Participants attended two clinic visits and were asked to wear a sensor-based activity monitor (Garmin Vivosmart^®^ HR Wrist Tracker) for 12 weeks. Twenty-six (72%) of participants accessed Vivametrica for the course of the study. The median number of steps at baseline and at study completion was 9329 (range 5406–18,228) and 10,181 (range 5398–21,401), respectively. There was no significant change in number of steps taken by the participants for the study period (Z = −1.086, *p* = 0.278). The women's responses to the three statements (related to using Vivametrica) are represented on bar graphs. Women's opinions were important to provide an understanding about how they realized the technology. Women did access Vivametrica. Women did not significantly increase their step count. However, these women were achieving beyond sedentary levels of activity (>5000 steps/day). Although the change in steps was not statistically significant, it represents a median increase in daily steps of 9%, which is clinically important.

## Introduction

Mortality rates from cardiovascular disease (CVD) have been substantially reduced for the past 60 years, with a reduction of 40% in the past decade.^1^ However, women continue to die prematurely of heart disease.^2,3^ A recent Canadian Heart and Stroke report^4^ suggests factors contributing to this problem are that women are “under-researched, underdiagnosed, undersupported, and underaware” of their CVD risk. New approaches need to be identified that will enable women to recognize modifiable risk factors (i.e., smoking, diabetes, physical inactivity, high blood pressure, high blood cholesterol, and obesity)^5^ and target their efforts toward prevention.

Physical activity is a modifiable CVD risk factor that is currently absent from cardiovascular risk scoring tools, including the “gold standard” Framingham risk score.^6^ CVD risk can be reduced by as much as 30% when individuals incorporate vigorous physical activity into their weekly routine.^7^ Furthermore, engaging in physical activity helps people reach their best/optimal health, both mentally and physically.^8^ Estimates suggest only 15% of Canadian adults achieve the minimum amount of daily recommended exercise.^1^ The World Health Organization has stated that physical activity for women can improve health and prevent disease. Despite this, physical inactivity remains more prevalent in women compared with men.^9^ Thus, innovative ways are needed to motivate behavioral changes for mitigating cardiovascular risk.

Wearable activity monitoring devices represent one potential approach for assisting women to identify modifiable risk factors for CVD. Sensor-based devices can capture personal data regarding physical activity, caloric expenditure, heart rate, blood pressure, and sleep. To date, using wearable technologies has been predominantly associated with monitoring physical activity. In recent years, products are now leveraging the data from wearable devices to further engage individuals and enhance awareness of health risk.

One such product is the web-based platform Vivametrica^™^, which combines physical activity data from wearable devices with a small amount of personal health information (e.g., date of birth, weight, height, and medical history) to provide estimates of risk for chronic diseases, including CVD. The calculated level of risk is then benchmarked against women of similar age in the Canadian general population.

There are potential advantages of using a wearable activity tracker (e.g., Garmin Vivosmart^®^ HR [GVWT]), and accessing Vivametrica to enable women to understand their CVD risk. Using Vivametrica does not require an appointment with a physician or receipt of blood work. Vivametrica is available anywhere (with access to the Wi-Fi/Internet), and is accessible using a smart phone, IPad^®^, tablet, or computer. Using passively collected information, Vivametrica provides personalized analysis of CVD risk, and actionable information for behavior change goal setting.

Our primary aim was to determine if women would access Vivametrica tools within the web-based platform. This would require women wearing the GVWT device that streams data to the platform and accessing the tools within the platform, such as the CVD risk assessment tool. Our secondary aims were to determine whether using the Vivametrica tools would lead women to increase their physical activity, as measured by their daily step counts, and to solicit women's opinions about using Vivametrica.

We used the technology acceptance model (TAM) as guidance to understand the characteristics and specifications required of new technology that provide the greatest potential for the new technology to be successfully used. Although an individual's perspectives related to quality, cost, mobility, availability, and cultural appeal do have an impact on use/adoption,^10–12^ it is the TAM that remains widely accepted and utilized when evaluating new technology for the goal of intention to use/adopt.^10,13^ We felt Vivametrica met this criterion but were keen to observe women's use and opinions.

## Methods

We used a prospective observational study design. After receipt of ethics approval from the Health Research Conjoint Ethics Board of the University of Calgary, participants were assessed at baseline (i.e., study entry) and 12 weeks thereafter. This time frame was chosen predominantly for feasibility. However, coauthor (C.T.-L.), an expert in use of web-based platforms, identified that “success” could be defined as a participant accessing Vivametrica a minimum of one time for a 12-week period.

### Recruitment

Participants were recruited through posters placed on public notice boards in general public places such as grocery stores, fitness centers, and drug stores, as well as at the University at which this study took place. Interested potential participants contacted the study coordinator. The coordinator reviewed the inclusion/exclusion criteria and booked potential participants for an enrolment baseline visit.

### Inclusion and exclusion criteria

All participants were required to be English speaking; able to attend two clinic visits; have access to a smart phone, iPad, tablet, or computer that could communicate with GVWT (wearable technology); have access to the Internet or Wi-Fi; and be able to provide informed consent. Potential participants were excluded from the study if they were unable to engage in physical activity, or cognitively unable to interact with the technology being used in the study.

### Data collection—baseline visit

After reiteration of the study purpose and expectations, all women provided written informed consent. Demographic data were collected using an investigator-developed form. Next, height, weight, and waist circumference were obtained using standardized measures,^13,14^ and body mass index (BMI) was calculated (BMI = weight [kg]/height [m^2^]).^15^ Blood pressure and resting heart rate were obtained by a registered nurse.^16^ Health-related data (e.g., CVD and diabetes history, smoking status) as well as previous experience of using wearable technology were also collected. CVD and diabetes were documented as “yes” if the participant had been diagnosed by a physician as having ischemic heart disease (problems with circulation to the heart, angina, or myocardial infarction) or diabetes (type I or type II). Smoking status was identified using Government of Canada guidelines.^17^

### Technology: wearable device and Vivametrica software

Participants were provided with a GVWT and instructions on proper usage. They were then asked to wear it on their dominant wrist for 24 h per day (unless charging the device) for a period of 12 weeks.

Participants were instructed on the use of Vivametrica, by the same resource person, from the Vivametrica company. The resource person also created a user account within the Vivametrica platform and a login ID number for each participant. Then, each participant's GVWT device was connected/synchronized to the Vivametrica platform, to allow Vivametrica to access this data from Garmin. Thereafter, each participant was asked to sign onto Vivametrica again, to ensure they could navigate the platform. Lastly, an instruction booklet (how to access/interact with Vivametrica), and an example of how to navigate Vivametrica was provided to each participant.

### Data flow

Data from the GVWT were streamed to the Vivametrica platform and isolated in a study file within the Vivametrica servers. Only the study investigators had access to the key that linked each participant to their Vivametrica ID. All data collected by Vivametrica were provided to the researcher at the end of the study (e.g., number of steps per day [physical activity level] and number of times that participants engaged with the Vivametrica platform [number of logins]).

### Data collection—12-week visit

All baseline measurements were repeated, and participants returned their GVWT. In addition, participants responded to a short Likert-type questionnaire designed to capture their impressions of Vivametrica. The statements were as follows: (1) The information form Vivametrica encourages me to do more physical activity; (2) Vivametrica is easy for me to use; and (3) Vivametrica increases my understanding of my cardiovascular risk. Participants were asked to respond to these statements using a 5-point scale [anchored with (1) strongly disagree to (5) strongly agree].

### Analysis

The study sample was characterized using descriptive statistics (means, percentages, as appropriate). The data of number of steps were analyzed from those participants who accessed Vivametrica. To evaluate whether there was a change in number of steps (indicator of physical activity) over time, a Wilcoxon signed rank test was used to compare the modal number of steps walked during the participants' first 14 days (T1) with the modal number of steps walked during the last 14 days (T2). This nonparametric test was used because there was a very large standard deviation identified when calculating the mean scores.

It is important to note that each participant had varying amounts of step data captured. For example, if a participant did not put their GVWT on after charging, or they did not synchronize their device with Vivametrica, their step data would not be captured. Therefore, for this analysis we used data from each participant's first 2 weeks of recorded step data and last 14 days of recorded step data. Given that 3 days of activity data in any given week satisfactorily estimates a person's weekly activity,^18^ we utilized step data if the participant had a recorded minimum of 3 days in each of the weeks being used for activity estimation. The women's responses to the three statements (related to using Vivametrica) are represented on bar graphs.

## Results

A sample of 38 women was recruited for an approximately 6-week period. Two women were excluded after enrolment as their electronic devices would not connect to Vivametrica. Thirty-six women who varied in age from 45 to 63 years ultimately participated in this study (Table 1). Most of the women were white, had postsecondary education, and were right-hand dominant. There was great variability in the range of participants' BMI, and the sample's mean BMI suggested overweight.^19^ Very few participants had a history of CVD or diabetes, and approximately one-third had a history of smoking. More than half (58%) of the participants indicated that they had previous experience with activity monitoring devices.

**Table 1.** Participant Characteristics, *n* = 36

Variable	Mean (SD)	Range
Age (years)	52.94 (4.89)	45–63
Height (cm)	161.99 (7.40)	148–178
Weight (kg)	74.07 (16.03)	49.1–127.6
Systolic (mmHg)	117.56 (11.64)	96–148
Diastolic (mmHg)	77.44 (8.93)	62–98
Pulse (bpm)	62.17 (7.77)	44–80
Waist circumference (cm)	94.10 (13.53)	75.5–142.0
BMI	28.35 (6.75)	19.64–55.59
Education	(%)	
High school	4 (11.1)	
College	13 (36.1)	
University	19 (52.8)	
Handedness		
Right	33 (91.7)	
Left	3 (8.3)	
Ethnicity		
White	33 (91.67)	
Other	3 (8.33)	
History of CVD	4 (10.5)	
History of diabetes	1 (2.6)	
Current smoker	1 (2.6)	
Ever smoker	12 (31.6)	

BMI, body mass index; CVD, cardiovascular disease; SD, standard deviation.

**FIG. 1.**
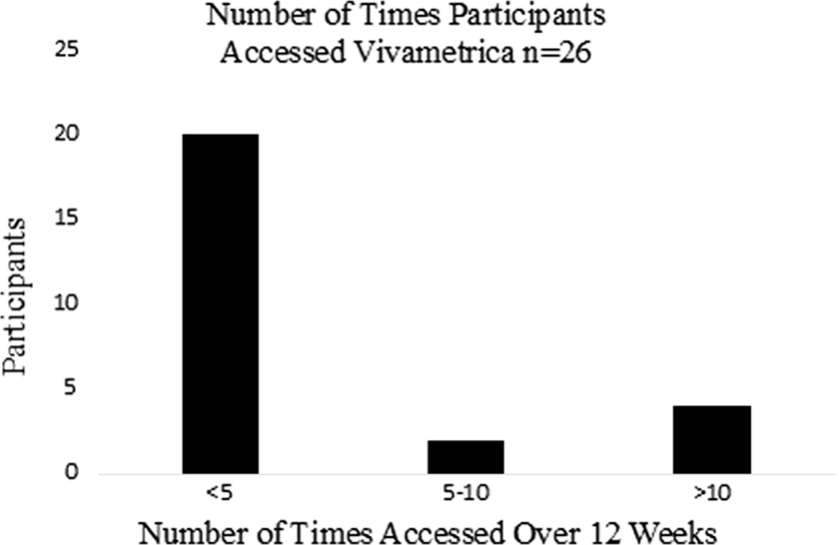
Number of times participants accessed Vivametrica^™^ for the study period.

Participants were categorized as accessing Vivametrica if they returned to the platform at least once after the initial access at the time of enrollment. Twenty-six (72%) of participants accessed Vivametrica for the course of the study (Fig. 1).

The modal number of steps at baseline and at 12 weeks were 5406 steps (range 5406–18,228 steps) and 8798 steps (range 5398–21,401 steps), respectively (Table 2).

**Table 2.** Average Daily Step Count for the 24 Participants Who Accessed Vivametrica and Had a Minimum of 3 Days of Step Data per Week

Participant No.	Time 1 (T1)	Time (T2)	Days between T1 and T2	Step difference from T1 to T2
001	5641	8736	63	3095 (+)
002	10,991	8543	56	2448 (−)
003	5406	6844	58	1438 (+)
006	7993	8622	56	629 (+)
007	9507	17,518	56	8011 (+)
009	6305	6752	56	447 (+)
013	11,504	10,672	45	832 (−)
014	8470	11,223	45	2753 (+)
015	7864	7572	46	292 (−)
017	10,453	9758	47	695 (−)
019	17,128	21,401	53	4273 (+)
020	10,847	8636	47	2211 (−)
021	8178	7366	45	812 (−)
022	6316	5927	47	389 (−)
023	7163	10,795	46	3632 (+)
024	5532	5398	47	134 (−)
025	9827	8997	43	830 (−)
026	14,507	13,345	44	1162 (−)
028	6550	9887	6	3337 (+)
029	6078	8338	47	2260 (+)
034	7109	9940	43	2831 (+)
036	18.228	15,809	42	2419 (−)
037	12.400	12,996	18	596 (+)
038	9907	9267	43	640 (−)

There was no significant change in number of steps taken by the participants who accessed Vivametrica for the 12-week period (Z = −1.086, *p* = 0.278).

**FIG. 2.**
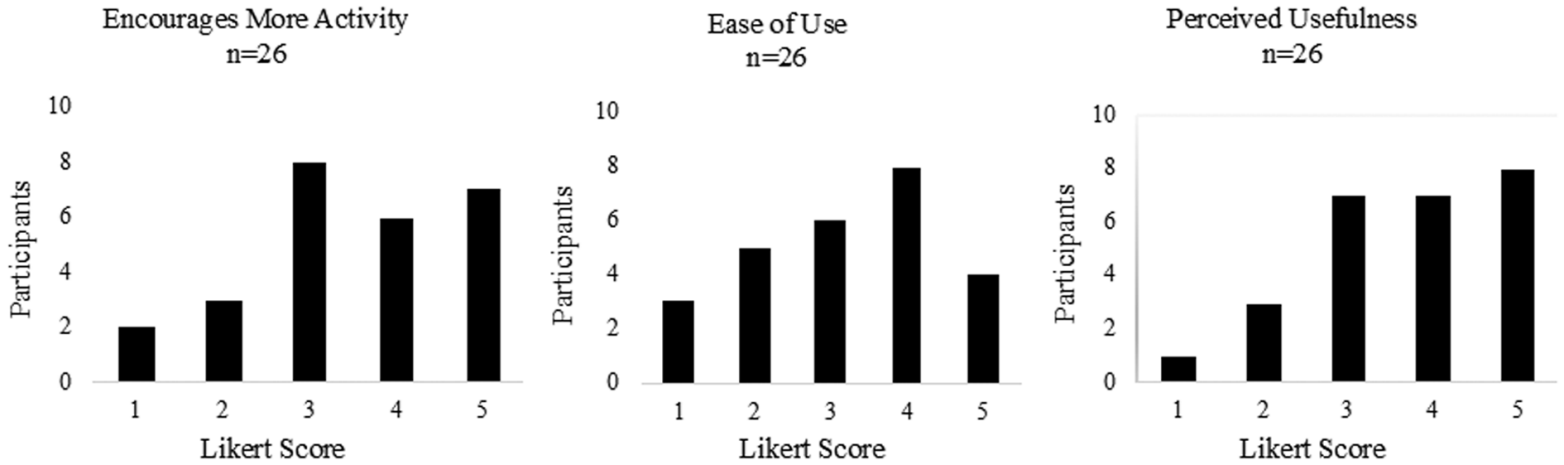
Participants' opinions about Vivametrica.

The participants who accessed Vivametrica responded to three questions soliciting their opinions about using Vivametrica (Fig. 2). Generally, the participants responded that using Vivametrica encouraged them to do more activity (81% [21/26] scored the tool ≥3). Most participants felt Vivametrica was easy to use (88% 23/26 scored the tool ≥3). Finally, participants scored Vivametrica lower in the category of usefulness (69% 18/26 scored the tool ≥3).

## Discussion

The goal of this study was to determine if women would interact with Vivametrica, benefit from using it (e.g., increase their step count), and identify if the technology was helpful to them. We found that the majority of the participants interacted with Vivametrica, the median step count was improved, and the majority had favorable impressions of the technology.

Although the majority of participants interacted with Vivametrica, 28% did not. Several participants indicated they did not recall how to/or even that they could access Vivametrica, despite having personalized instruction and an instruction booklet available. This may be the result of introducing two pieces of technology (Vivametrica and GVWT) at the same time. The introduction of two types of technology seemed to create confusion, for some women, about using Vivametrica versus the application for Garmin on the participants' mobile devices.

Researchers have noted from the literature, that men have a natural affinity with technology and women have a fear of technology.^20^ Lohan and Faulkner^21^ emphasized that technology and gender are both socially constructed, and one cannot be completely understood without the other. van Oost^22^ posited that women are taught/conditioned to be intimidated by technology and men are encouraged to embrace technology as evidenced by the concept of “gender scripting.” Yet, more recent study^23^ suggested that older men and women utilize information and communication technology. This suggests that over time, and exposure to technology, women as well as men, become more comfortable using technology. This bodes well as the population for this study being 45–64 years of age.

There was no statistically significant increase in median step count of the course of the study. There are two possible reasons for this finding. The participant group was generally healthy (4% history of CVD) and none of the participants were sedentary. Thus, as the Vivametrica system was accessed, participants generally were likely not to have received encouragement to increase their activity.

It is worth noting that all 26 women who accessed Vivametrica were already achieving beyond sedentary levels of activity (>5000 steps/day).^24^ The women who interacted with the technology increased their activity by 9%. This is clinically important^25–29^ as an increase in physical activity can reduce your risk of dying from leading causes of death (heart disease and some cancers).^9,30^ The Center for Disease Control elaborates on physical activity being available to everyone and each individual can gain health benefits from physical activity irrespective of age, ethnicity, shape, or size.^30^

Most participants had favorable impression of the technology, although for some, there was a lack of acceptance. The TAM^31,32^ is useful in guiding the understanding of how this technology was utilized and appreciated by women. As described in the TAM, the first determinant of technology adoption is “ease of use” and the second determinant is an individual's “perceived usefulness.” The participants responded more favorably to ease of use than perceived usefulness. Participants' did not score “perceived usefulness” as high as ease of use on the Likert Scale.

The introduction of two pieces of technology (Vivametrica and GVWT) seemed to create confusion about using Vivametrica versus the application for Garmin on the participants' mobile devices. It may have been beneficial to provide the participants with GVWT for a period of time and then introduce Vivametrica, so the participants had time to become familiar with the purpose/difference of the GVWT.

The TAM forms the foundation for this study describing the importance of “perceived ease of use” and “perceived usefulness” as key components when individuals are considering adoption of new technology.^27^ Predominantly, women liked the idea of having a resource such as Vivametrica particularly in regard to cardiovascular/health risk scores. However, some participants felt disappointed that they could not navigate/understand the platform/scores, and, thus, the potential benefits were lost.

Wang et al.^33^ reported perceived confidence in ability to use technology has a positive effect on an individual's perception of usefulness of the technology. Buchanan and Lockton^34^ suggested that how the information is received (what data are available) and the individual's motives are factors when considering engagement, suggesting if an individual's perceived needs are not addressed, engagement may not be successful. In this study, participants described not having enough information to understand the various components of the GVWT and Vivametrica. Several participants would have liked the option of having more tutorials and training. Also, accessing the computer to realize their health scores was not convenient. Women were wanting Vivametrica to be available on their smart phones, similar to GVWT. Rooksby et al.,^35^ described the importance of considering personal preferences when considering technology.

### Strengths and limitations

The majority of women in this study accessed the technology, increased their weekly step count, and had a favorable opinion of the technology. However, we had a small sample size and followed them for a limited amount of time. Several participants indicated they did not recall how to/or even that they could access Vivametrica, despite having personalized instruction and an instruction booklet available. Introducing two types of technology at the same time created confusion for women and possibly reduced the overall number of women who accessed Vivametrica.

## Conclusion

This is a small descriptive study and is not generalizable to the population. However, this study provides a glimpse of how women would interact with a novel cardiovascular platform, such as Vivametrica. Lastly, it is importance to be guided by a model such as the TAM to support understanding the criteria for new technology to be adopted.

## Abbreviations Used

BMI: body mass index
CVD: cardiovascular disease
TAM: technology acceptance model
